# Radiation-induced lung toxicity in mice irradiated in a strong magnetic field

**DOI:** 10.1371/journal.pone.0205803

**Published:** 2018-11-16

**Authors:** Ashley E. Rubinstein, Skylar Gay, Christine B. Peterson, Charles V. Kingsley, Ramesh C. Tailor, Julianne M. Pollard-Larkin, Adam D. Melancon, David S. Followill, Laurence E. Court

**Affiliations:** 1 Department of Diagnostic and Interventional Imaging, UTHealth McGovern Medical School, Houston, Texas, United States of America; 2 Department of Radiation Physics, The University of Texas MD Anderson Cancer Center, Houston, Texas, United States of America; 3 Department of Biostatistics, The University of Texas MD Anderson Cancer Center, Houston, Texas, United States of America; 4 Department of Imaging Physics, The University of Texas MD Anderson Cancer Center, Houston, Texas, United States of America; ENEA Centro Ricerche Casaccia, ITALY

## Abstract

Strong magnetic fields affect radiation dose deposition in MRI-guided radiation therapy systems, particularly at interfaces between tissues of differing densities such as those in the thorax. In this study, we evaluated the impact of a 1.5 T magnetic field on radiation-induced lung damage in C57L/J mice. We irradiated 140 mice to the whole thorax with parallel-opposed Co-60 beams to doses of 0, 9.0, 10.0, 10.5, 11.0, 12.0, or 13.0 Gy (20 mice per dose group). Ten mice per dose group were irradiated while a 1.5 T magnetic field was applied transverse to the radiation beam and ten mice were irradiated with the magnetic field set to 0 T. We compared survival and noninvasive assays of radiation-induced lung damage, namely respiratory rate and metrics derived from thoracic cone-beam CTs, between the two sets of mice. We report two main results. First, the presence of a transverse 1.5 T field during irradiation had no impact on survival of C57L/J mice. Second, there was a small but statistically significant effect on noninvasive assays of radiation-induced lung damage. These results provide critical safety data for the clinical introduction of MRI-guided radiation therapy systems.

## Introduction

Magnetic resonance imaging (MRI)-guided radiation therapy (MRIgRT) systems can be used to perform real-time imaging during radiation therapy, offering more precise patient-tailored treatments. There exist various MRIgRT systems, both in the prototype stage and in current clinical use [1–4]. In most of these systems, the strong magnetic field is always present when delivering radiation. Systems with a lower MRI magnetic field strength of 0.35T have the disadvantage of lower signal-to-noise and reduced contrast enhancement. Systems with higher magnetic field strengths have superior image quality. However, magnetic fields affect radiation dose deposition, and this effect is amplified with higher magnetic field strengths, especially if the MRI’s magnetic field is oriented perpendicular to the radiation beam [5–8].

Inside the MRI’s magnetic field, the Lorentz force acts on dose-depositing secondary electrons. This creates perturbations in the resulting dose distribution, such as a reduced build-up distance; a laterally shifted, asymmetric penumbra; and hot and cold spots at interfaces between tissues of different densities [9]. One area of concern in MRIgRT is the thorax, where Monte Carlo simulations have shown an increase in dose at the transition from higher density soft tissue to lower density lung tissue, as well as a decrease in dose when transitioning from lung tissue to soft tissue [8, 10]. Before MRIgRT systems can be used clinically, these effects should be thoroughly investigated.

There is a need for preclinical *in vivo* experiments that will allow us to study dose perturbations at tissue interfaces and help us better understand the biological consequences of magnetic-field-induced radiation dose effects. If magnetic fields do influence radiobiological damage, we expect to see the effects in the lungs because magnetic field effects cause larger dose perturbations in inhomogeneous target areas. Furthermore, the lungs have a relatively high sensitivity to radiation, and complications are quite common after irradiation of the thoracic region [11]. Radiation-induced complications in the lungs are characterized by an acute syndrome, radiation pneumonitis, and subsequent chronic radiation fibrosis [12, 13]. Lung cancer patients often undergo radiation therapy, and because of the high radiosensitivity of the lungs, these patients are at high risk for radiation-induced pneumonitis and fibrosis [14].

Radiation-induced lung injury has unique clinical, histopathologic, and radiographic characteristics; therefore, there are several techniques that can be used to measure this damage in mice. Studies have shown a correlation between radiation-induced lung toxicity, such as a reduction in blood oxygenation, and post-irradiation changes in the lungs using thoracic imaging [15–20]. Respiratory rate has also shown to be correlated with histological changes in the lungs after irradiation [21] and is often used as a non-invasive technique to measure radiation-induced lung damage in mice [17, 18, 22–31].

Existing techniques to measure respiratory rate require expensive equipment and that mice be removed from their regular environment. We aimed to improve the reproducibility and reliability of our work by developing a novel technique which was low cost and allowed for measurements to be made on mice that remained in their housing. In our study, we assessed whether measurements of respiratory rate, as well as metrics derived from free-breathing CBCT imaging, were predictive of survival from radiation-induced lung toxicity. We then evaluated the effect of a 1.5 T field on radiation-induced lung damage by irradiating C57L/J mice to the whole thorax with parallel-opposed Co-60 beams in the presence of a transverse 1.5 T field. We compared the survival data and lung toxicity metrics of these mice with those of C57L/J mice irradiated in no magnetic field.

## Methods

### Mouse irradiation

This work was approved by the University of Texas MD Anderson Cancer Center Institutional Animal Care and Use Committee. A mouse model was used in this study because the lung radiosensitivity of mice has been well studied, a larger sample size was achievable than if a large-animal model had been used, and it is more feasible to generate a magnetic field strength of 1.5 T surrounding a mouse than a larger animal. Monte Carlo simulations and film measurements have demonstrated that a 1.5 T magnetic field and a Co-60 beam can produce dose perturbations in mice comparable to those seen in human MRIgRT simulations [32, 33].

This study included 140 8-week-old female C57L/J mice, in accordance with the guidelines provided by The University of Texas MD Anderson Cancer Center IACUC. The mice were housed in microisolator cages on a ventilated rack system within a specific pathogen-free facility. C57L/J mice were used because they are susceptible to both acute pneumonitis and subsequent chronic fibrosis at post-irradiation timepoints similar to humans [11, 17]. Other strains of mice that are commonly used in lung radiation sensitivity studies, such as CBA/J, CBA/Ca, C3H/HeJ, C57BL/6J, C57BL/Cbi, CBBF1, WHT, and TO mice, develop significant pleural effusions that cause more respiratory-related morbidities and mortalities than do pneumonitis and fibrosis [17]. C57L/J mice rarely develop this condition [17, 34, 35]. Furthermore, these mice were shown to have a dose response for the incidence of severe pneumonitis that most closely resembles that of non-human primates and humans [30].

**Fig 1:**
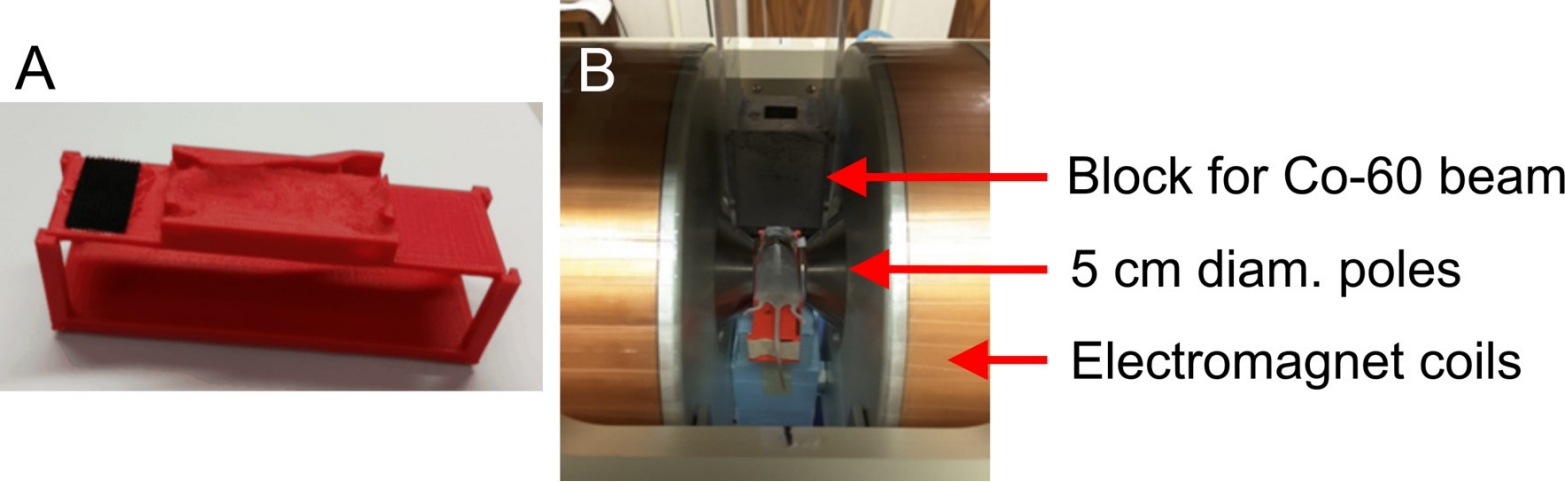
The set-up for the irradiation of the C57L/J mice. A) The 3D-printed holder and B) the set-up for a Co-60 PA irradiation between the poles of the H-frame electromagnet.

The mice were anesthetized with 1.5%-2.5% isoflurane and oxygen at a flow rate of 1.5 L/min during their set-up and irradiation. The mice were positioned in a 3D-printed holder between the poles of an H-frame dipole electromagnet (3472–70, GMW Associates, San Carlos, CA, USA) (Fig 1). The 3D-printed holder was created using a whole-body CBCT of an 8-week-old C57L/J mouse to increase the reproducibility of positioning so that the entire thorax could be irradiated while sparing the surrounding organs. The pole faces of the electromagnet were 5 cm in diameter, and the gap between the poles was 3.5 cm. The strength and homogeneity of the magnetic field were verified with a Gaussmeter (GM-2, AlphaLab, Inc., Salt Lake City, UT, USA).

The mice underwent irradiation of the whole thorax with parallel-opposed 2.1 cm x 2.1 cm AP/PA Co-60 beams (9.0, 10.0, 10.5, 11.0, 12.0, or 13.0 Gy) in either a 1.5 T field (n = 60) or a 0 T field (n = 60). Twenty control mice did not undergo radiation. Mice were housed with other mice at their dose level (five mice per cage). Each week, a different dose group was irradiated (n = 20 per dose group). The first set of mice in each dose group was irradiated with the electromagnet turned off (n = 10). The second set was irradiated with the electromagnet turned on and the field set at 1.5 T (n = 10).

Respiratory rate, lung density, and healthy lung volume measurements were obtained monthly until the end of the study at 8 months post-irradiation to assess the severity of radiation-induced lung damage. Two days before irradiation, baseline respiratory rate measurements were obtained while the mice were sleeping in their cages; the next day, they were imaged using free-breathing CBCT to obtain baseline lung density and healthy lung volume measurements. Respiratory rate measurements were always made the day before the mice were imaged so that the measurements would not be affected by the anesthesia used during imaging.

### Respiratory motion tracking and respiratory rate calculation

Mice were recorded while sleeping in their standard laboratory cages with a 30 fps, 1080p webcam (HD Pro Webcam C920, Logitech, Lausanne, Switzerland). On average, the mice were recorded for 15.84 (10.17–21.18) min. Motion due to respiration was tracked using computer vision techniques. The open-source software that was created for the respiratory rate calculation, as well as instructions on how to use the software, can be found in Appendix A. For each mouse, we identified at least five different 20-second clips of video in which the mouse was sleeping and breathing regularly (no gasping, sniffing, or pauses in respiration). On the first frame of the clip, contours were drawn on the portion of each mouse where respiration caused the mouse’s fur to move. Within each contour, up to 10 corners (regions of an image that contain high spatial frequency information) were detected using OpenCV’s function for Shi-Tomasi corner detection. These corners were then tracked through the frames of the 20-second video using the Lucas-Kanade method for estimating optical flow. The output of the motion tracking was a waveform of the mouse’s respiratory motion. The amplitude of the waveform is dependent on the distance from the camera to the points in the fur that are being tracked, as well as the angle at which the measurements are made. The frequency of the waveform, however, is independent of these factors; this is the property of interest as it represents the mouse’s respiratory rate.

To validate our respiratory rate measurement technique, we compared the motion tracking respiratory rates to visual measurements in three 20-second video clips of 20 mice. The visual measurements were made by slowing the video clips down to one quarter of the speed and counting the breaths taken. In 98% of the validation measurements, the calculated respiratory rates matched the visually measured respiratory rates to within 5 bpm.

### Lung density and healthy lung volume measurements

Free-breathing CBCT imaging was used to measure lung density and healthy lung volume before and after irradiation. Acquisitions were made on the X-RAD 225Cx system (Precision X-Ray; North Branford, CT), which was operated at 60 kVp, 4 mA, and 3 rpm for a total scan length of 20 seconds. Each mouse was anesthetized with 1.5%-2.5% isoflurane and oxygen at a flow rate of 1.5 L/min. The mice were positioned in the 3D-printed holder described above during imaging. Radiopaque numbered labels were 3D printed using PLA filament. For each image acquisition, the timepoint number was placed on the left side of the mouse and the mouse identification number was placed on the right side. This was done to reduce errors in assigning measurements to specific timepoints for specific mice.

Healthy lung tissue was delineated using auto-threshold contouring (0–0.7 g/cm^3^) in a treatment planning system (Pinnacle3, Philips Medical Systems, Andover, MA), and the volume of the delineated lungs was calculated using the same software. The density of the lungs was measured using ImageJ software [36] with 7 regions of interest (ROIs) distributed in fixed locations throughout the lung. The ROIs were 1.5 mm x 1.5 mm in the apex of the lung (4 ROIs) and 2.4 mm x 2.4 mm in the base of the lung (3 ROIs). The ROIs were placed in the same positions in each mouse’s lungs for each timepoint to ensure that we would not bias the results with our ROI placement (see Appendix B)

Mice were monitored daily and were euthanized by isoflurane overdose and cervical dislocation when there was evidence of severe respiratory distress (e.g., lethargic, hunched back, and ungroomed fur) and more than a 20% loss of weight, or at the end of the study (8 months post-irradiation).

### Data analysis

Before comparing radiation-induced lung damage between the 1.5 T group of mice and the 0 T group of mice, we first assessed the correlation between survival and our non-invasive assays of lung damage. We compared the respiratory rates, lung densities, and healthy lung volumes of mice that survived until the end of the study and mice that died from radiation-induced lung injury using Welch’s unequal variances *t*-test. We used measurements made 5 months post-irradiation, as mice had developed radiation-induced pneumonitis by this point. If the mouse did not survive until the 5-month timepoint, we used measurements made at the 4-month timepoint.

We next used univariate Cox proportional hazards models to evaluate the association between survival and each of the three predictor variables: respiratory rate, lung density, and healthy lung volume. Finally, we dichotomized the mice into normal and abnormal groups for each of the lung injury metrics, where normal values were defined as no more than 3 standard deviations above the mean values of the control mice at the 5-month timepoint (or minus 3 standard deviations for the healthy lung volume metric, as healthy lung volume decreases post-irradiation), and all values beyond this threshold were defined as abnormal.

Finally, Spearman’s correlation was estimated among the measurements of respiratory rate, lung density, and healthy lung volume at 5 months post-irradiation. The two assumptions that underpin Spearman’s correlation are that the variables are measured on an ordinal or continuous scale and that there is a monotonic relationship between them [37]. These assumptions held for each of the three comparisons that were made.

To compare the lung damage of mice irradiated in a transverse 1.5 T field and mice irradiated in no magnetic field, we first evaluated the survival curves for each of the dose groups (and for both the 1.5 T and 0 T fields) and compared them using a log-rank test. We next compared the rate of normal versus abnormal respiratory rates, lung densities, and healthy lung volumes between the 1.5 T and 0 T groups of mice. Non-linear regression (GraphPad Prism, San Diego, CA, USA) was used to fit the Hill equation to dose-response curves for each of the three responses. Dose-response relationships were investigated, and differences between the median effective doses (ED50) at 5 months post-irradiation were compared using an extra sum-of-squares F-test.

## Results

### Correlation between survival and radiation-induced lung injury metrics

For 8 of the 140 mice in this study, their cause of death was not related to radiation-induced lung damage (e.g., 5 mice died before irradiation); therefore, they were not included in the results. The respiratory rates and lung densities at the measurement timepoint were significantly higher (p<0.001) in mice that died from radiation-induced lung injury (n = 71) than in those that survived until the end of the study (n = 61). The healthy lung volumes were significantly lower (p<0.001). The respiratory rates, lung densities, and healthy lung volumes of the two groups can be found in Table 1.

**Table 1:** The mean ± SEM values of non-invasive assays of radiation-induced lung damage in 132 mice 5 months after irradiation.

Assay	Survival status	
	Survived (n = 61)	Died (n = 71)
Respiratory rate	153 bpm ± 3 bpm	191 bpm ± 5 bpm
Lung density	0.54 g/cm^3^ ± 0.01 g/cm^3^	0.77 g/cm^3^ ± 0.02 g/cm^3^
Healthy lung volume	0.51 cm^3^ ± 0.01 cm^3^	0.32 cm^3^ ± 0.02 cm^3^

We found that the respiratory rate, lung density, and healthy lung volume measurements were all significantly predictive of survival (p<0.0001). Table 2 shows the Cox proportional hazards model parameter estimates, p-values, and hazard ratios of each of the predictor variables.

**Table 2:** Cox proportional hazards model of non-invasive assay measurements in irradiated mice.

Predictor variable	Coefficient estimate	Hazard ratio (95% CI)
Respiratory rate (bpm)	0.027	1.03 (1.02–1.03)
Lung density (g/cm^3^)	15.94	8.38e + 06 (4.65e + 05–1.51e + 08)
Healthy lung volume (cm^3^)	-8.913	1.35e - 04 (2.01e - 05–0.90e - 04)

p<0.0001 for all predictor variables

The values used to dichotomize the mice into normal and abnormal groups for each of the lung injury metrics are shown in Table 3. For the respiratory rate measurements, there were 33 mice in the normal group and 99 mice in the abnormal group. For the lung density measurements, there were 62 mice in the normal group and 70 mice in the abnormal group. For the healthy lung volume measurements, there were 53 mice in the normal group and 79 mice in the abnormal group.

**Table 3:** Control group values used to dichotomize mice into normal and abnormal groups.

Lung injury metric	Mean of control mice ± SD	Cut-off for abnormality
Respiratory rate	142 bpm ± 16 bpm	190 bpm
Lung density	0.474 g/cm^3^ ± 0.055 g/cm^3^	0.639 g/cm^3^
Healthy lung volume	0.580 cm^3^ ± 0.053 cm^3^	0.421 cm^3^

**Fig 2:**
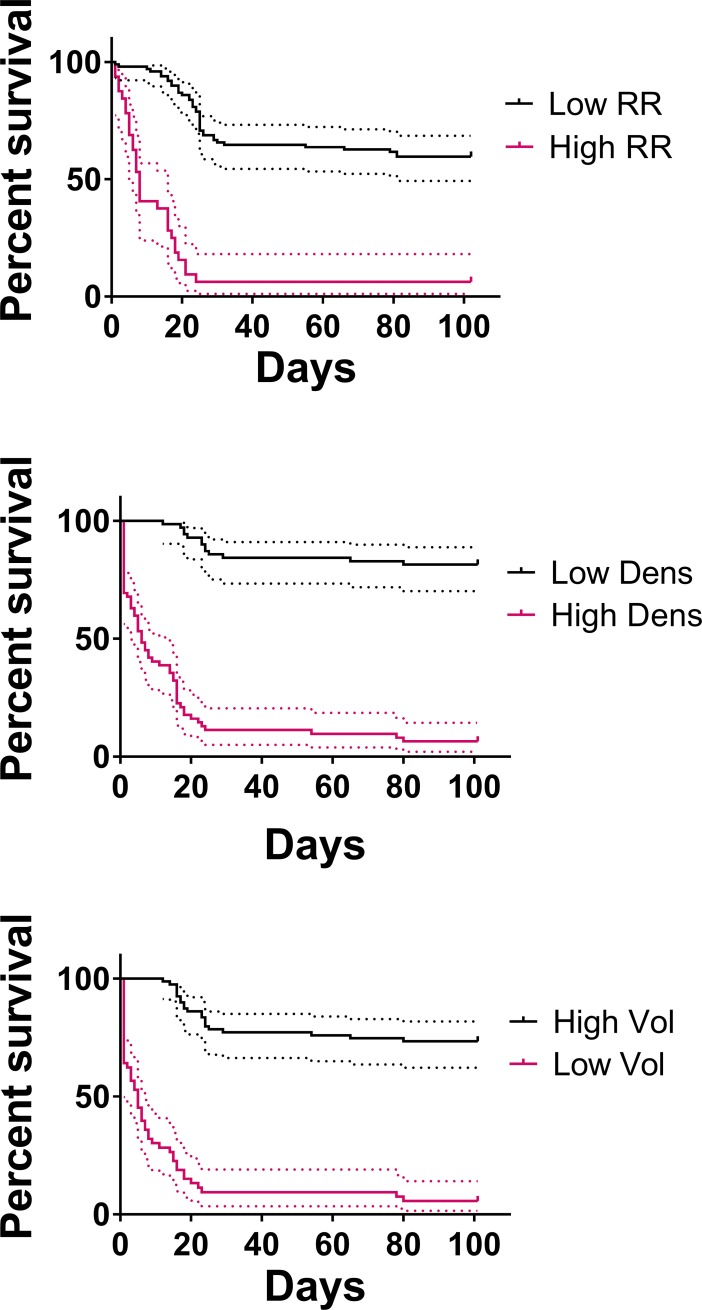
Survival after the 5-month measurement (or the 4-month measurement if mice did not survive for 5 months), separated by the average control mice values at that timepoint ± three standard deviations.

Survival from the measurement timepoint (5 months post-irradiation, or 4 in mice that did not survive for 5) was compared between mice with normal and abnormal values for each of the three metrics (Fig 2). The survival curves were significantly different for each of the comparisons (p<0.0001).

The correlation between respiratory rate and lung density measurements was significant (r = 0.6827, p<0.0001), as were the correlations between respiratory rate and healthy lung volume measurements (r = -0.6098, p<0.0001) and healthy lung volume and lung density measurements (r = -0.8424, p<0.0001). This data is shown in Appendix C.

### Survival: 1.5 T vs. 0 T

**Fig 3:**
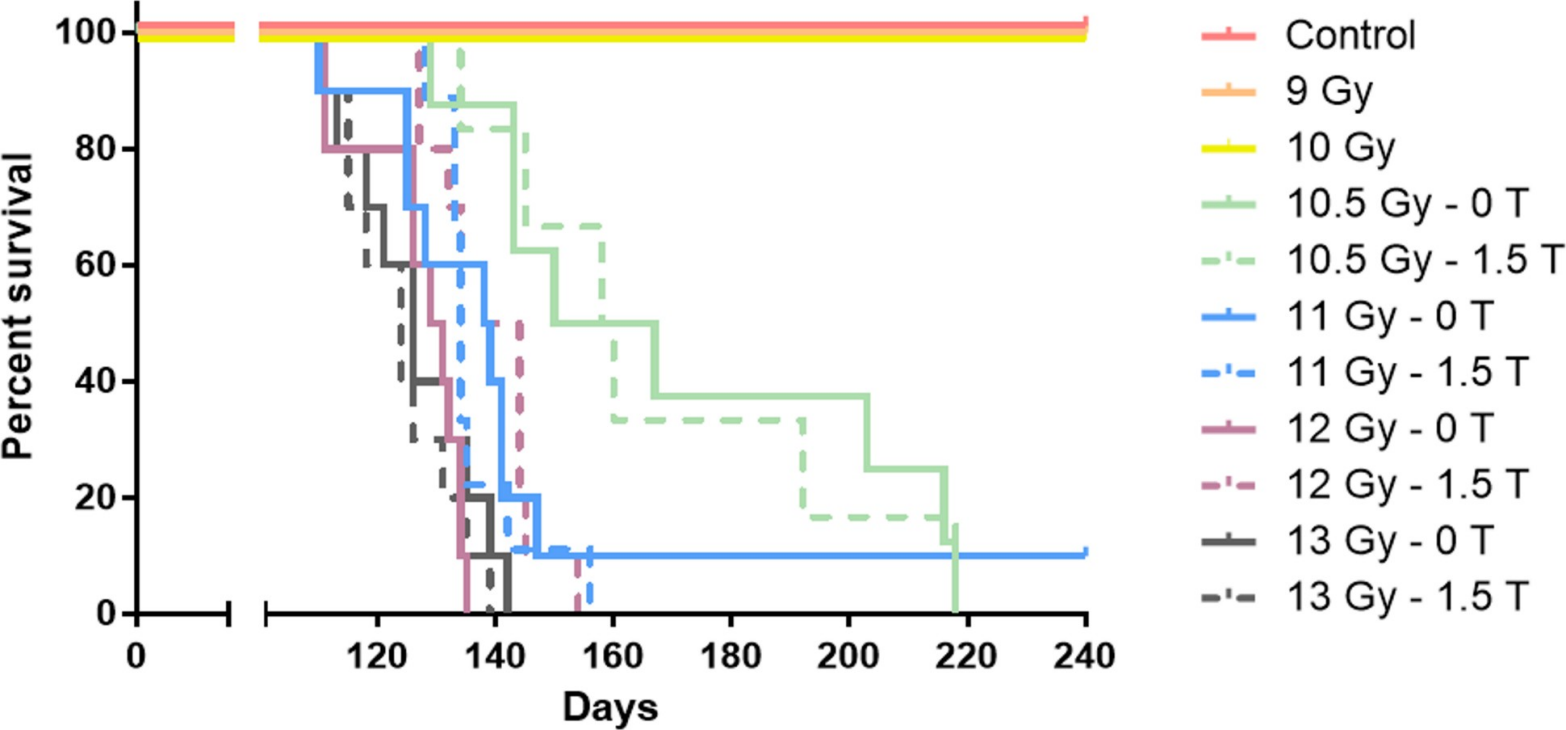
Survival of mice in all dose groups. Curves for both the 0 T and 1.5 T groups of mice are shown for doses of 10.5 Gy-13.0 Gy.

The 1.5 T magnetic field had little to no effect on survival for all of the dose groups (Fig 3). All mice in the 9.0 Gy, 10.0 Gy, and control groups survived until the end of the study, regardless of whether they had been irradiated in a 1.5 T or 0 T field. There was no significant difference in survival between mice in the 1.5 T and 0 T groups for doses of 10.5 Gy, 11.0 Gy, and 13.0 Gy. There was a small but statistically significant difference in survival between mice in the 1.5 T and 0 T groups for 12.0 Gy (adjusted log-rank p = 0.03). However, the lack of difference for higher and lower doses and the overlap of the 12.0 Gy/1.5 T curve with both 11.0 Gy curves lead us to believe that this result should not be over-interpreted given the context of the overall experiment. Moreover, a multivariate Cox regression analysis including both dose and magnetic field strength as predictors of survival across our entire data set showed that dose had a highly statistically significant association (p<0.0001), while the association for magnetic field strength was not significant.

### Severity of radiation-induced lung injury: 1.5 T vs. 0 T

**Fig 4:**
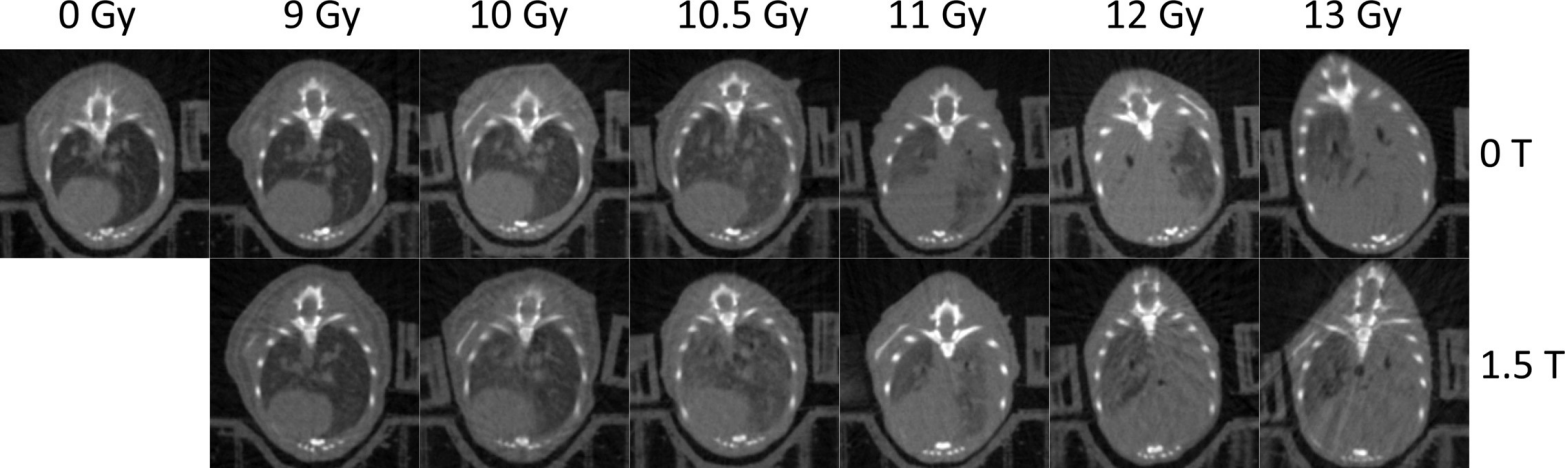
Axial CBCTs of the lungs of mice in each dose group for the 0 T and 1.5 T magnetic fields at 5 months post-irradiation. The severity of radiation-induced lung injury (an increase in lung density and a decrease in healthy lung volume) increases with increasing dose for both the 0 T and 1.5 T fields.

**Fig 5:**
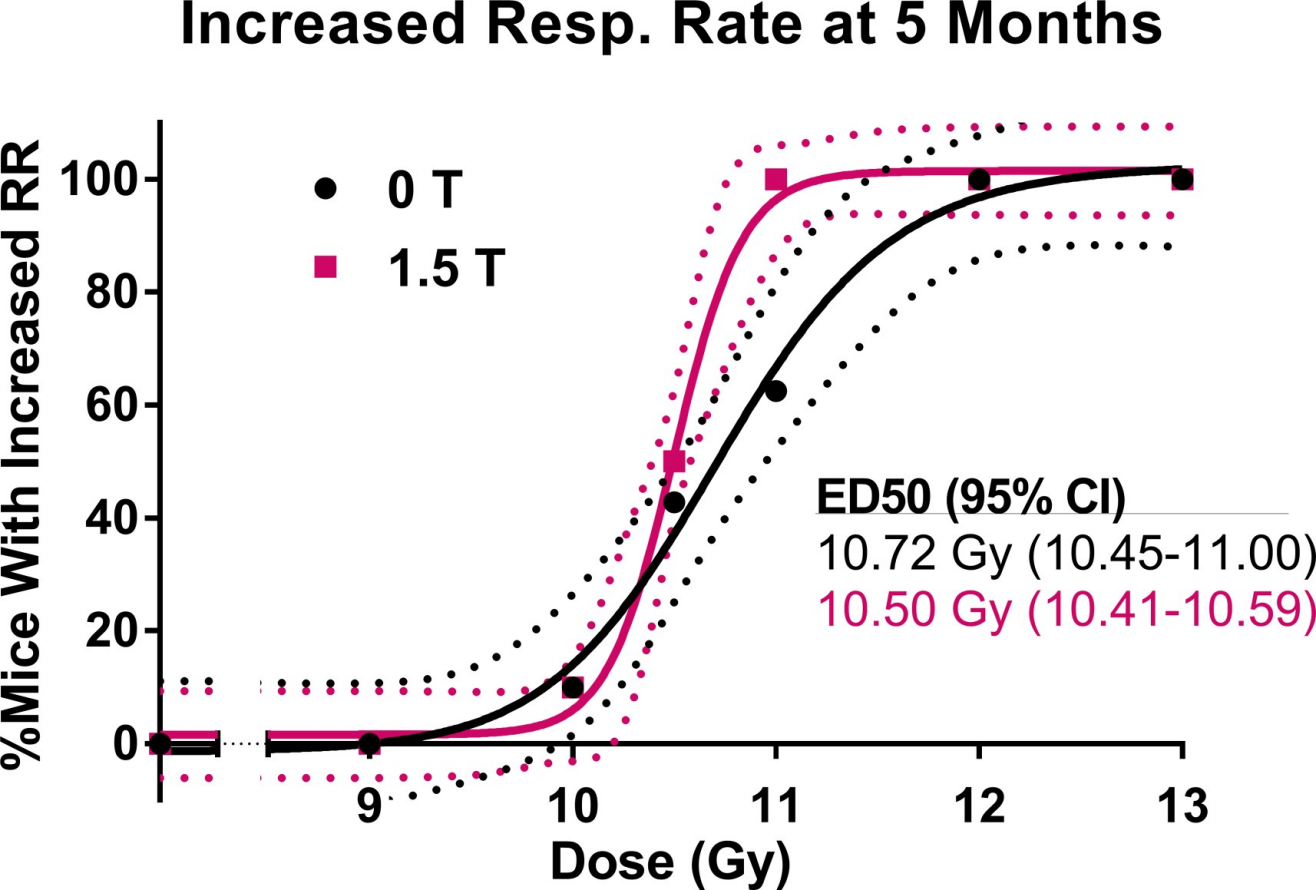
The dose-response curves for mice irradiated to 0, 9.0, 10.0, 10.5, 11.0, 12.0, or 13.0 Gy in either a 0 T or 1.5 T magnetic field. The dose response is the percentage of mice in each dose/magnetic field group with an increased respiratory rate by 5 months post-irradiation. An increased respiratory rate was defined as being more than three standard deviations above the mean of the respiratory rate of control mice at 5 months post-irradiation.

**Fig 6:**
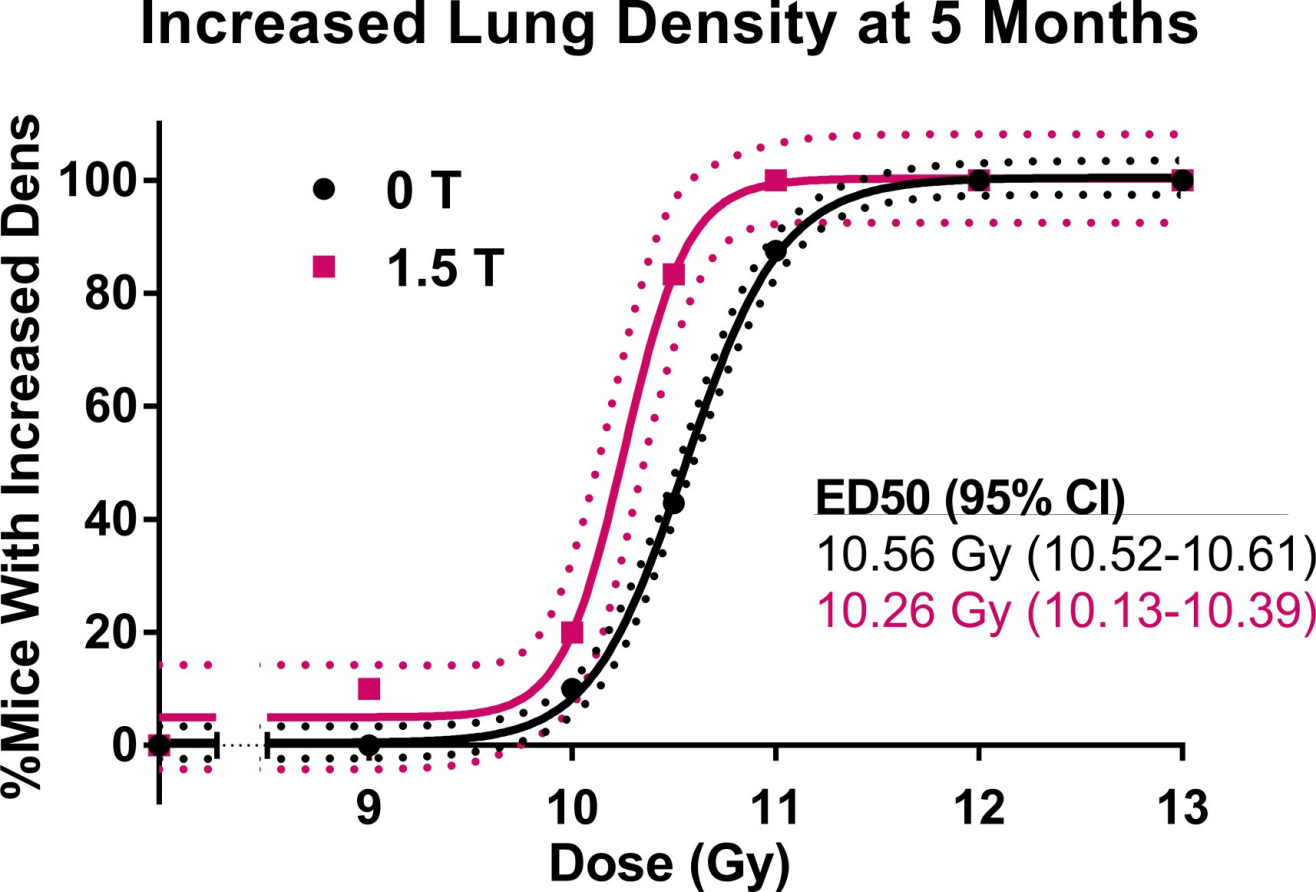
The dose-response curves for mice irradiated to 0, 9.0, 10.0, 10.5, 11.0, 12.0, or 13.0 Gy in either a 0 T or 1.5 T magnetic field. The dose response is the percentage of mice in each dose/magnetic field group with an increased lung density by 5 months post-irradiation. An increased lung density was defined as being more than three standard deviations above the mean of the lung density of control mice at 5 months post-irradiation.

**Fig 7:**
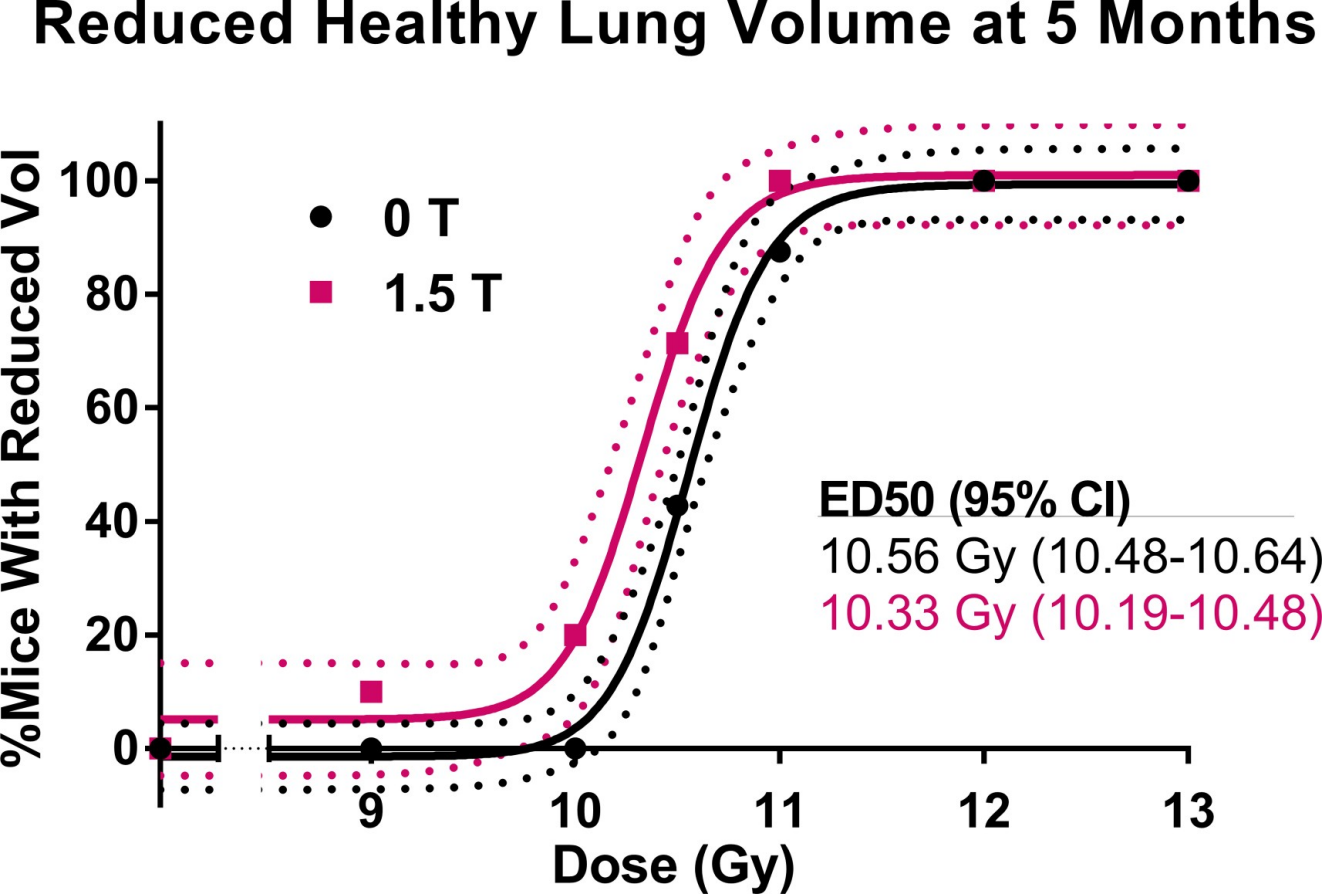
The dose-response curves for mice irradiated to 0, 9.0, 10.0, 10.5, 11.0, 12.0, or 13.0 Gy in either a 0 T or 1.5 T magnetic field. The dose-response is the percentage of mice in each dose/magnetic field group with a reduced healthy lung volume by 5 months post-irradiation. A reduced healthy lung volume was defined as being lower than three standard deviations below the mean of the healthy lung volume of control mice at 5 months post-irradiation.

CBCTs of mice in each dose group for the 0 T and 1.5 T fields are shown in Fig 4. The dose-response relationships for the metrics of increased respiratory rate, increased lung density, and reduced healthy lung volume can be seen in Fig 5, Fig 6 and Fig 7, respectively. Increased respiratory rate and increased lung density were defined as values larger than the means of the control mice values plus three standard deviations. A reduced healthy lung volume was defined as a value lower than the mean healthy lung volume of control mice minus three standard devations. For each of the three metrics, the 1.5 T dose-response curves were shifted slightly to the left of the 0 T curves. With increased respiratory rate as the response, the mice that had been irradiated in the presence of a 1.5 T magnetic field had an ED50 that was 2% lower than that of the mice that had been irradiated in no magnetic field (p = 0.0332). With increased lung density as the response, the mice that had been irradiated in the presence of a 1.5 T magnetic field had an ED50 that was 3% lower than that of the mice that had been irradiated in no magnetic field (p = 0.0017). With reduced healthy lung volume as the response, the mice that had been irradiated in the presence of a 1.5 T magnetic field had an ED50 that was 2% lower than that of the mice that had been irradiated in no magnetic field (p = 0.0033). In summary, all three assays showed a small but statistically significant increase in response to radiation in the presence of a magnetic field.

## Discussion

In this study, we found that respiratory rate, lung density, and healthy lung volume measurements obtained 5 months post-irradiation were all highly predictive of survival after irradiation of the whole thorax. We dichotomized the mice into abnormal and normal groups based on their measurements 5 months post-irradiation and found that survival was significantly lower in the abnormal groups for each of the three metrics.

The survival trends and relatively high lung radiation sensitivity of the C57L/J mice in this study was consistent with the findings of other groups [17, 26, 30, 31, 34, 35, 38]. We compared mice irradiated in the presence of a 1.5 T magnetic field to those irradiated in the absence of a magnetic field and found that the 1.5 T field had little to no effect on survival. The dose to which the mice were irradiated was a much stronger predictor of survival. When we assessed the effect of irradiation in a 1.5 T field on non-invasive measurements of radiation-induced lung damage, however, we found that there was a significant effect on the ED50 at 5 months post-irradiation for each of the three measured responses (an increased respiratory rate, an increased lung density, and a reduced healthy lung volume). For each of the three responses, the ED50 for the 1.5 T group was smaller than that for the 0 T group. While these differences were small (2%-3%), they were consistent between the different measurements.

Previous studies have investigated the correlation between post-irradiation changes seen in lung imaging and radiation-induced lung damage. El-Khatib, *et al* [15] used CT to monitor changes in lung density after irradiation, which they correlated to histological changes due to radiation-induced pneumonitis. Plathow, *et al* [16] used CT to detect increases in lung density, intralobular opacity, and fibrotic strands to monitor the development of radiation-induced lung fibrosis. Other research groups, when comparing the response to thoracic radiation in various strains of mice, measured post-irradiation changes in lung density and volume on both micro-CT and MRIs [17–19]. The results of our study demonstrated that post-irradiation changes in lung density and healthy lung volume were significantly predictive of survival (p<0.0001), and therefore support the continued utility of imaging as a non-invasive tool to measure radiation-induced lung damage.

Respiratory rate is most commonly measured using either an unrestrained single-chambered whole-body plethysmograph or a restrained double-chambered plethysmograph [39]. While plethysmography is appealing because it does not rely on anesthesia, intubation, tracheostomy, or thoracotomy, it is costly and requires the mouse to be taken out of its standard environment, which introduces stress and could affect the measurement [40, 41]. Another existing technique for measuring respiratory rate is the use of a pulse oximeter, which can be clipped onto the tail of an anesthetized mouse or placed around the neck of a conscious or anesthetized mouse (MouseOx Plus, STARR Life Sciences, Oakmont, PA). While these techniques are minimally invasive, they have the disadvantages of being costly, having a low throughput, being time intensive, having questionable reproducibility, and causing stress to the animal (which in turn affects the measurement). In the study herein, we developed a completely non-invasive technique for measuring respiratory rate in mice while they are asleep in standard laboratory cages. This technique is low cost: the only piece of equipment needed is a camera capable of recording at least 30 fps, and we have made the measurement software open source.

Our results demonstrated that the measurements of respiratory rate, lung density, and healthy lung volume at 5 months post-irradiation were correlated with each other. While each metric is valuable on its own, this correlation demonstrates that if a study is limited in terms of time, funds, or equipment, it is possible to use only one of these techniques to assess radiation-induced lung injury.

One limitation of our study was that mice underwent irradiation of the whole thorax, whereas in MRIgRT treatments, a smaller portion of the patient’s lungs would be expected to be irradiated. Another limitation was that we assessed the effect of magnetic-field-induced dose perturbations on radiation-induced pneumonitis, but did not assess the effect on subsequent fibrosis. Only mice from the 0 Gy, 9.0 Gy, and 10.0 Gy dose groups survived until the end of the study. This was not a sufficient number of mice to draw any definite conclusions about the effect of a magnetic field on radiation-induced fibrosis.

Our finding that there was no significant difference in survival between mice irradiated in a 1.5 T magnetic field and mice irradiated in the absence of a magnetic field supports the idea that MRIgRT is safe for use in the thoracic region. However, our finding that the 1.5 T field affected measurements of respiratory rate, lung density, and healthy lung volume suggests that further investigation is warranted. Although the strong transverse magnetic field had no effect on survival, its effect on our assays of radiation-induced lung damage implies that MRIgRT in the thoracic region could potentially affect patients’ quality of life.

It has been hypothesized that dose hot spots and dose cold spots in MRIgRT treatment plans can be eliminated with parallel-opposed fields or Monte-Carlo-based IMRT treatment planning [5, 6, 8, 42–44]. While this is certainly possible in the planning stage, it is not necessarily the case in patients, who have heterogeneous, malleable tissue that can change in position over time. Care should be taken in introducing MRIgRT to the clinic, especially for treatments in regions with inhomogeneous tissue. It is important to rigorously test new technologies in the preclinical stage before they are introduced to the clinic. Although a technique may be theoretically possible, we should be cautious in the clinical introduction of a technology before it has been thoroughly investigated.

## References

[1] Jaffray DA, Carlone MC, Milosevic MF, Breen SL, Stanescu T, Rink A, et al A facility for magnetic resonance-guided radiation therapy. Semin Radiat Oncol. 2014;24(3):193–5. 10.1016/j.semradonc.2014.02.012 . 24931091 10.1016/j.semradonc.2014.02.012

[2] Keall PJ, Barton M, Crozier S, Australian MRI-Linac Program icfII, Illawarra Cancer Care Centre, Liverpool Hospital, Stanford University, Universities of Newcastle, Queensland, Sydney, W.stern Sydney, and Wollongong. The Australian magnetic resonance imaging-linac program. Semin Radiat Oncol. 2014;24(3):203–6. 10.1016/j.semradonc.2014.02.015 . 24931094 10.1016/j.semradonc.2014.02.015

[3] Mutic S, Dempsey JF. The ViewRay system: magnetic resonance-guided and controlled radiotherapy. Semin Radiat Oncol. 2014;24(3):196–9. 10.1016/j.semradonc.2014.02.008 . 24931092 10.1016/j.semradonc.2014.02.008

[4] Kerkmeijer LG, Fuller CD, Verkooijen HM, Verheij M, Choudhury A, Harrington KJ, et al The MRI-Linear Accelerator Consortium: Evidence-Based Clinical Introduction of an Innovation in Radiation Oncology Connecting Researchers, Methodology, Data Collection, Quality Assurance, and Technical Development. Front Oncol. 2016;6:215 Epub 2016/10/13. 10.3389/fonc.2016.00215 ; PubMed Central PMCID: PMCPMC5061756. 27790408 10.3389/fonc.2016.00215 PMC5061756

[5] Kirkby C, Stanescu T, Rathee S, Carlone M, Murray B, Fallone BG. Patient dosimetry for hybrid MRI-radiotherapy systems. Medical Physics. 2008;35(3):1019 10.1118/1.2839104 18404937 10.1118/1.2839104

[6] Kirkby C, Murray B, Rathee S, Fallone BG. Lung dosimetry in a linac-MRI radiotherapy unit with a longitudinal magnetic field. Med Phys. 2010;37(9):4722–32. 10.1118/1.3475942 . 20964190 10.1118/1.3475942

[7] Yang YM, Geurts M, Smilowitz JB, Sterpin E, Bednarz BP. Monte Carlo simulations of patient dose perturbations in rotational-type radiotherapy due to a transverse magnetic field: a tomotherapy investigation. Med Phys. 2015;42(2):715–25. 10.1118/1.4905168 ; PubMed Central PMCID: PMCPMC4297282. 25652485 10.1118/1.4905168 PMC4297282

[8] Raaijmakers AJ, Raaymakers BW, Lagendijk JJ. Magnetic-field-induced dose effects in MR-guided radiotherapy systems: dependence on the magnetic field strength. Phys Med Biol. 2008;53(4):909–23. 10.1088/0031-9155/53/4/006 . 18263948 10.1088/0031-9155/53/4/006

[9] Raaymakers BW, Raaijmakers AJE, Kotte ANTJ, Jette D, Lagendijk JJW. Integrating a MRI scanner with a 6 MV radiotherapy accelerator: dose deposition in a transverse magnetic field. Physics in Medicine and Biology. 2004;49(17):4109–18. 10.1088/0031-9155/49/17/019 15470926 10.1088/0031-9155/49/17/019

[10] Raaijmakers AJ, Raaymakers BW, Lagendijk JJ. Integrating a MRI scanner with a 6 MV radiotherapy accelerator: dose increase at tissue-air interfaces in a lateral magnetic field due to returning electrons. Phys Med Biol. 2005;50(7):1363–76. 10.1088/0031-9155/50/7/002 . 15798329 10.1088/0031-9155/50/7/002

[11] Williams JP, Brown SL, Georges GE, Hauer-Jensen M, Hill RP, Huser AK, et al Animal models for medical countermeasures to radiation exposure. Radiation research. 2010;173(4):557–78. 10.1667/RR1880.1 ; PubMed Central PMCID: PMC3021126. 20334528 10.1667/RR1880.1 PMC3021126

[12] Gagliardi G, Bjohle J, Lax I, Ottolenghi A, Eriksson F, Liedberg A, et al Radiation pneumonitis after breast cancer irradiation: analysis of the complication probability using the relative seriality model. Int J Radiat Oncol Biol Phys. 2000;46(2):373–81. . 10661344 10.1016/s0360-3016(99)00420-4

[13] Davis SD, Yankelevitz DF, Henschke CI. Radiation effects on the lung: clinical features, pathology, and imaging findings. AJR American journal of roentgenology. 1992;159(6):1157–64. 10.2214/ajr.159.6.1442375 . 1442375 10.2214/ajr.159.6.1442375

[14] Rodrigues G, Lock M, D'Souza D, Yu E, Van Dyk J. Prediction of radiation pneumonitis by dose—volume histogram parameters in lung cancer—a systematic review. Radiotherapy and oncology: journal of the European Society for Therapeutic Radiology and Oncology. 2004;71(2):127–38. 10.1016/j.radonc.2004.02.015 . 15110445 10.1016/j.radonc.2004.02.015

[15] El-Khatib E, Sharplin J, Battista J. The density of mouse lung in vivo following X irradiation. Int J Radiat Oncol Biol Phys. 1983;9(6):853–8. . 6863058 10.1016/0360-3016(83)90011-1

[16] Plathow C, Li M, Gong P, Zieher H, Kiessling F, Peschke P, et al Computed tomography monitoring of radiation-induced lung fibrosis in mice. Investigative radiology. 2004;39(10):600–9. . 15377939 10.1097/01.rli.0000138134.89050.a5

[17] Jackson IL, Vujaskovic Z, Down JD. Revisiting strain-related differences in radiation sensitivity of the mouse lung: recognizing and avoiding the confounding effects of pleural effusions. Radiation research. 2010;173(1):10–20. 10.1667/RR1911.1 ; PubMed Central PMCID: PMC2818983. 20041755 10.1667/RR1911.1 PMC2818983

[18] Jackson IL, Vujaskovic Z, Down JD. A further comparison of pathologies after thoracic irradiation among different mouse strains: finding the best preclinical model for evaluating therapies directed against radiation-induced lung damage. Radiation research. 2011;175(4):510–18. 10.1667/RR2421.1 ; PubMed Central PMCID: PMC3110676. 21338245 10.1667/RR2421.1 PMC3110676

[19] Down JD, Yanch JC. Identifying the high radiosensitivity of the lungs of C57L mice in a model of total-body irradiation and bone marrow transplantation. Radiation research. 2010;174(2):258–63. 10.1667/RR2149.1 . 20681792 10.1667/RR2149.1

[20] Bickelhaupt S, Erbel C, Timke C, Wirkner U, Dadrich M, Flechsig P, et al Effects of CTGF Blockade on Attenuation and Reversal of Radiation-Induced Pulmonary Fibrosis. J Natl Cancer Inst. 2017;109(8). 10.1093/jnci/djw339 . 28376190 10.1093/jnci/djw339

[21] Travis EL, Down JD, Holmes SJ, Hobson B. Radiation pneumonitis and fibrosis in mouse lung assayed by respiratory frequency and histology. Radiation research. 1980;84(1):133–43. . 7454976

[22] Travis EL, Vojnovic B, Davies EE, Hirst DG. A plethysmographic method for measuring function in locally irradiated mouse lung. Br J Radiol. 1979;52(613):67–74. 10.1259/0007-1285-52-613-67 . 427355 10.1259/0007-1285-52-613-67

[23] Rappaport DS, Niewoehner DE, Kim TH, Song CW, Levitt SH. Uptake of carbon monoxide by C3H mice following X irradiation of lung only or total-body irradiation with 60Co. Radiation research. 1983;93(2):254–61. . 6337382

[24] Down JD, Collis CH, Jeffery PK, Steel GG. The effects of anesthetics and misonidazole on the development of radiation-induced lung damage in mice. Int J Radiat Oncol Biol Phys. 1983;9(2):221–6. . 6833025 10.1016/0360-3016(83)90103-7

[25] Down JD, Easton DF, Steel GG. Repair in the mouse lung during low dose-rate irradiation. Radiotherapy and oncology: journal of the European Society for Therapeutic Radiology and Oncology. 1986;6(1):29–42. . 3520698 10.1016/s0167-8140(86)80107-4

[26] Franko AJ, Sharplin J. Assessment of radiation-induced lung injury in mice using carbon monoxide uptake: correlation with histologically visible damage. Radiation research. 1993;133(2):245–51. . 8438066

[27] Liao ZX, Travis EL, Tucker SL. Damage and morbidity from pneumonitis after irradiation of partial volumes of mouse lung. Int J Radiat Oncol Biol Phys. 1995;32(5):1359–70. 10.1016/0360-3016(94)00660-D . 7635776 10.1016/0360-3016(94)00660-D

[28] van Rongen E, Travis EL, Thames HD Jr. Repair rate in mouse lung after clinically relevant radiation doses per fraction. Radiation research. 1995;141(1):74–8. . 7997517

[29] Heinzelmann F, Jendrossek V, Lauber K, Nowak K, Eldh T, Boras R, et al Irradiation-induced pneumonitis mediated by the CD95/CD95-ligand system. J Natl Cancer Inst. 2006;98(17):1248–51. 10.1093/jnci/djj335 . 16954477 10.1093/jnci/djj335

[30] Jackson IL, Xu PT, Nguyen G, Down JD, Johnson CS, Katz BP, et al Characterization of the dose response relationship for lung injury following acute radiation exposure in three well-established murine strains: developing an interspecies bridge to link animal models with human lung. Health physics. 2014;106(1):48–55. 10.1097/HP.0b013e3182a32ccf . 24276549 10.1097/HP.0b013e3182a32ccf

[31] Jackson IL, Xu P, Hadley C, Katz BP, McGurk R, Down JD, et al A preclinical rodent model of radiation-induced lung injury for medical countermeasure screening in accordance with the FDA animal rule. Health physics. 2012;103(4):463–73. 10.1097/HP.0b013e31826386ef ; PubMed Central PMCID: PMC3604892. 22929472 10.1097/HP.0b013e31826386ef PMC3604892

[32] Rubinstein AE, Liao Z, Melancon AD, Guindani M, Followill DS, Tailor RC, et al Technical Note: A Monte Carlo study of magnetic-field-induced radiation dose effects in mice. Med Phys. 2015;42(9):5510–6. 10.1118/1.4928600 . 26328998 10.1118/1.4928600 PMC5148183

[33] Rubinstein A, Tailor R, Melancon A, Pollard J, Guindani M, Followill D, et al Magnetic-Field-Induced Dose Effects in a Mouse Lung Phantom:Monte Carlo and Experimental Assessments. American Association of Physicists in Medicine. 2016;43(6):1 10.1118/1.4958143

[34] Sharplin J, Franko AJ. A quantitative histological study of strain-dependent differences in the effects of irradiation on mouse lung during the intermediate and late phases. Radiation research. 1989;119(1):15–31. . 2756106

[35] Sharplin J, Franko AJ. A quantitative histological study of strain-dependent differences in the effects of irradiation on mouse lung during the early phase. Radiation research. 1989;119(1):1–14. . 2756101

[36] Abràmoff MD, Magalhães PJ, Ram SJ. Image processing with ImageJ. Biophotonics international. 2004;11(7):36–42.

[37] Hauke JK, Tomasz Comparison of Values of Pearson’s And Spearman’s Correlation Coefficients. Quaestiones Geographicae. 2011;30(2).

[38] Franko AJ, Sharplin J, Ghahary A, Barcellos-Hoff MH. Immunohistochemical localization of transforming growth factor beta and tumor necrosis factor alpha in the lungs of fibrosis-prone and "non-fibrosing" mice during the latent period and early phase after irradiation. Radiation research. 1997;147(2):245–56. . 9008217

[39] DeLorme MP, Moss OR. Pulmonary function assessment by whole-body plethysmography in restrained versus unrestrained mice. J Pharmacol Toxicol Methods. 2002;47(1):1–10. . 12387933 10.1016/s1056-8719(02)00191-0

[40] Pibiri F, Nelson M, Guidotti A, Costa E, Pinna G. Decreased corticolimbic allopregnanolone expression during social isolation enhances contextual fear: A model relevant for posttraumatic stress disorder. Proc Natl Acad Sci U S A. 2008;105(14):5567–72. Epub 2008/04/07. 10.1073/pnas.0801853105 ; PubMed Central PMCID: PMCPMC2291140. 18391192 10.1073/pnas.0801853105 PMC2291140

[41] Tuli JS, Smith JA, Morton DB. Stress measurements in mice after transportation. Lab Anim. 1995;29(2):132–8. 10.1258/002367795780740249 . 7602999 10.1258/002367795780740249

[42] Raaijmakers AJ, Hardemark B, Raaymakers BW, Raaijmakers CP, Lagendijk JJ. Dose optimization for the MRI-accelerator: IMRT in the presence of a magnetic field. Phys Med Biol. 2007;52(23):7045–54. 10.1088/0031-9155/52/23/018 . 18029992 10.1088/0031-9155/52/23/018

[43] Bol GH, Lagendijk JJ, Raaymakers BW. Compensating for the impact of non-stationary spherical air cavities on IMRT dose delivery in transverse magnetic fields. Phys Med Biol. 2015;60(2):755–68. 10.1088/0031-9155/60/2/755 . 25559321 10.1088/0031-9155/60/2/755

[44] Menten MJ, Fast MF, Nill S, Kamerling CP, McDonald F, Oelfke U. Lung stereotactic body radiotherapy with an MR-linac—Quantifying the impact of the magnetic field and real-time tumor tracking. Radiotherapy and oncology: journal of the European Society for Therapeutic Radiology and Oncology. 2016 10.1016/j.radonc.2016.04.019 . 27165615 10.1016/j.radonc.2016.04.019 PMC4936791

